# Utilization of Neuroimaging Techniques in the Differential Diagnosis of Very Late-Onset Schizophrenia and Neurodegenerative Disorders

**DOI:** 10.7759/cureus.80482

**Published:** 2025-03-12

**Authors:** Nicolas Biaggi, Jacklyn Potts, Alfred Torres, Melissa L Verzura, Ashley S Bourne, Jose Cruz

**Affiliations:** 1 Psychiatry, Mount Sinai Medical Center, Miami Beach, USA; 2 Dr. Kiran C. Patel College of Osteopathic Medicine, Nova Southeastern University, Fort Lauderdale, USA; 3 Medical School, Florida International University, Herbert Wertheim College of Medicine, Miami, USA; 4 Psychiatry/Neurology, Mount Sinai Medical Center, Miami Beach, USA

**Keywords:** brain fdg pet, dementia with lewy body, geriatric psychiatry, neuro-imaging, schizophrenia and other psychotic disorders

## Abstract

The emergence of psychiatric symptoms in late life presents a diagnostic challenge due to their overlap with neurodegenerative disorders. This case details a 66-year-old male with acute psychosis, initially raising concerns for an underlying neurodegenerative-related process. Comprehensive evaluation, including neuroimaging and laboratory studies, excluded neurodegeneration, leading to a diagnosis of very late-onset schizophrenia. The patient demonstrated clinical improvement with an atypical antipsychotic, while an acetylcholinesterase inhibitor was discontinued due to the absence of neurodegenerative pathology. This case highlights the critical role of thorough diagnostic assessment in differentiating primary psychiatric disorders from neurodegenerative conditions in older adults.

## Introduction

Numerous psychiatric disorders, including schizophrenia, and neurodegenerative conditions such as Alzheimer’s disease (AD) and dementia with Lewy bodies (DLB) may manifest with hallucinations and delusions in the elderly. While schizophrenia predominantly presents in early adulthood, it can also emerge as late-onset (post-45 years) or very late-onset schizophrenia (post-65 years) [1]. According to the Diagnostic and Statistical Manual of Mental Disorders, Fifth Edition (DSM-5), a diagnosis of schizophrenia necessitates the presence of delusions, hallucinations, or disorganized speech, accompanied by disorganized behavior or negative symptoms, for a minimum duration of six months [2].

Alzheimer’s disease, the most prevalent neurodegenerative disorder, may exhibit similar neuropsychiatric symptoms, particularly during the middle stages of the disease, with these manifestations intensifying in parallel with cerebral degeneration. Dementia with Lewy bodies is also frequently characterized by prominent visual hallucinations and delusions, with a higher prevalence of delusions in DLB (49%) compared to AD (31%), despite their persistence in both conditions [3].

The inherent overlap in symptomatology among psychiatric diagnoses poses a considerable challenge to clinicians. Patients presenting with psychosis, hallucinations, and delusions may be ascribed to multiple diagnostic categories, contingent upon the temporal progression of symptoms and associated clinical features. Psychosis is broadly defined as “the presence of delusions, hallucinations without insight, or both” [4], a definition widely endorsed in clinical practice.

Differentiating between these conditions necessitates a meticulous and comprehensive psychiatric evaluation, incorporating an extensive patient history to establish a primary psychiatric diagnosis while systematically excluding a broad spectrum of medical etiologies. This diagnostic process is particularly complex in the geriatric population, which is disproportionately affected by neurodegenerative diseases, particularly Alzheimer’s disease. The advent of neuroimaging techniques, including brain MRI and fluorodeoxyglucose-positron emission tomography (FDG-PET) scans, has significantly enhanced the diagnostic accuracy for neurodegenerative diseases, thereby providing valuable insights in cases with overlapping psychiatric symptoms [5].

We present a case who initially presented to the psychiatric inpatient unit and was later referred to the Wien Center for Alzheimer’s Disease and Memory Disorders at Mount Sinai Medical Center for the exclusion of neurodegenerative pathology due to him exhibiting clinical features consistent with very late-onset schizophrenia.

## Case presentation

A 66-year-old male with a medical history of hypertension and no prior psychiatric diagnoses was brought to the emergency department (ED) by his family under an ex parte order issued by a judge for urgent evaluation due to acute psychosis. Upon arrival, the patient underwent a comprehensive medical assessment by the ED physician and was deemed medically stable for psychiatric evaluation. Initial laboratory investigations, including CBC, basic metabolic panel (BMP), urinalysis, and urine culture, were all within normal limits.

Collateral information obtained from the patient’s daughter revealed that approximately one year prior to presentation, the patient retired, moved into her residence, and began exhibiting decreased social interaction. Ten months prior to the current presentation, the patient experienced his first "episode," characterized by bizarre behavior and obsessions lasting one to two weeks, which resolved spontaneously. Shortly thereafter, he had a second "episode," marked by withdrawal, reduced communication with family members, and subsequent spontaneous resolution after one week. Two weeks prior to this ED visit, the patient began displaying signs of self-neglect, bizarre and paranoid delusions, visual hallucinations of "zombies," and agitation. His daughter described him as "not listening, not eating, not sleeping, and wanting to leave the house to stay in his car all day." Additionally, he exhibited unusual behavior, such as refusing to touch anything that was not blue.

During the initial psychiatric evaluation, the patient presented with a disheveled appearance, a euthymic mood, and a normal affect. Although guarded, his speech was of normal rhythm and volume. He did not show any evidence of abnormal movements or extrapyramidal symptoms. He endorsed paranoia and persecutory delusions involving an individual in a red Mercedes-Benz whom he believed was blocking his driveway and attempting to control or kidnap him. He denied experiencing symptoms consistent with depression, anxiety, or mania.

The patient’s background information revealed that he was born in Haiti and had resided in South Florida for the past 26 years. He had worked as a certified nursing assistant, predominantly on night shifts, until his retirement one year prior to this presentation. He had four children and, at the time of evaluation, lived with one of his daughters. His medical history was notable for hypertension and a previous deep vein thrombosis (DVT), for which he was taking aspirin 81 mg daily. The family history of psychiatric illness included a grandmother who reportedly passed away with dementia and a mother who allegedly experienced pseudodementia following the death of her niece. The patient denied any history of substance use, and his urine toxicology screen was negative.

Given the clinical presentation, the decision was made to admit the patient to the inpatient psychiatric unit, where he was initiated on olanzapine 2.5 mg nightly. The patient demonstrated a positive response to the medication, with daily improvements in his symptoms, and was discharged after six days of hospitalization. During the admission, it was also noted that the patient had experienced worsening memory over the past year, leading to a referral for memory evaluation at the Wien Center for Alzheimer’s disease.

One month post-discharge, the patient underwent a neuropsychiatric evaluation at the Wien Center, where he scored 21/30 on the Mini-Mental State Examination (MMSE), losing points in recall, serial sevens, and orientation. The test was administered with the assistance of a Creole-speaking interpreter. His family reported that his basic and complex activities of daily living (ADLs) remained intact. Following this evaluation, he was prescribed donepezil 5 mg daily in addition to continuing olanzapine 2.5 mg nightly. A brain MRI was ordered, and a follow-up was scheduled for four weeks later.

**Figure 1:**
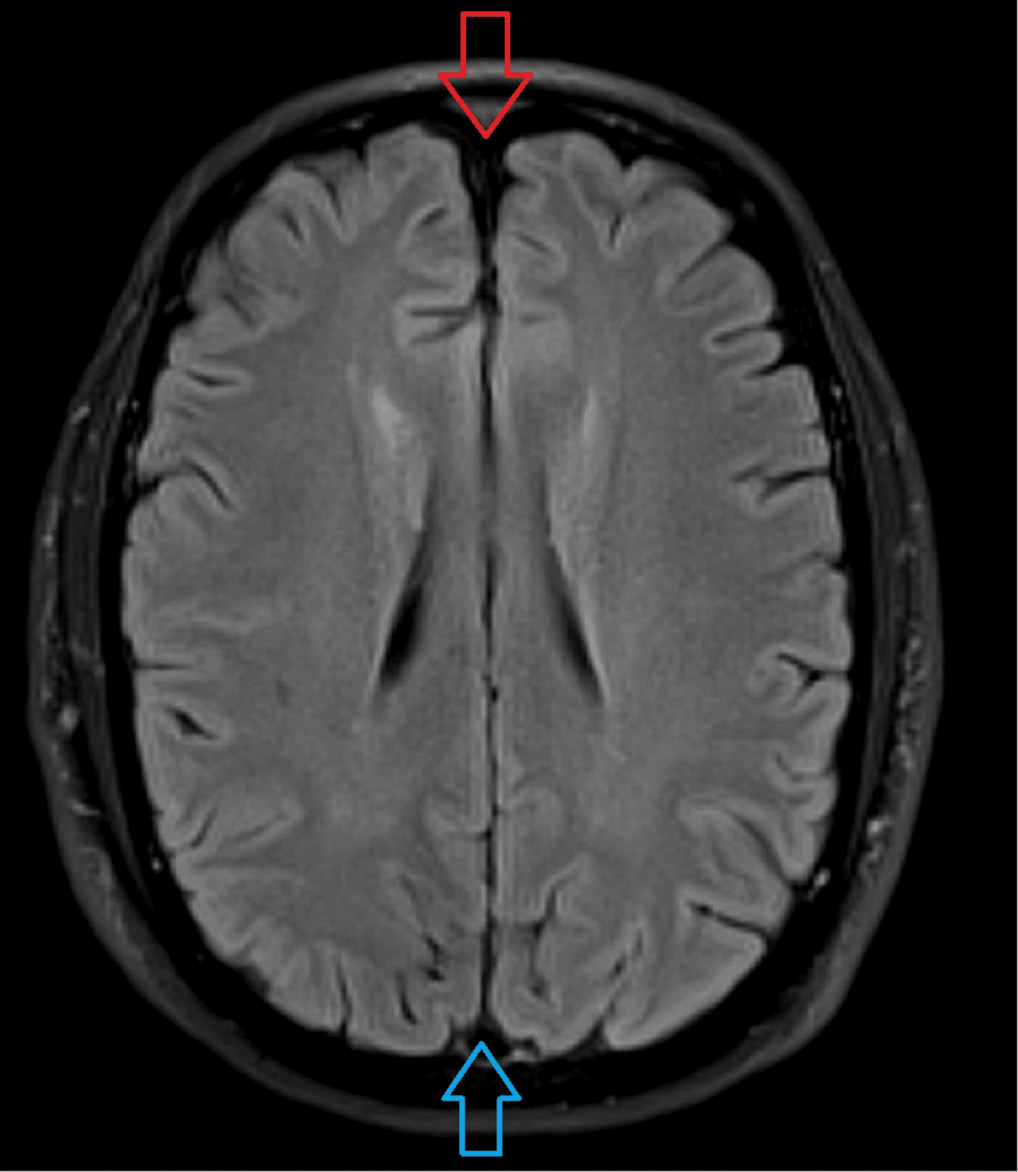
Axial MRI of the brain No significant neocortical atrophy seen in the frontal (red arrow) and parietal lobes (blue arrow).

**Figure 2:**
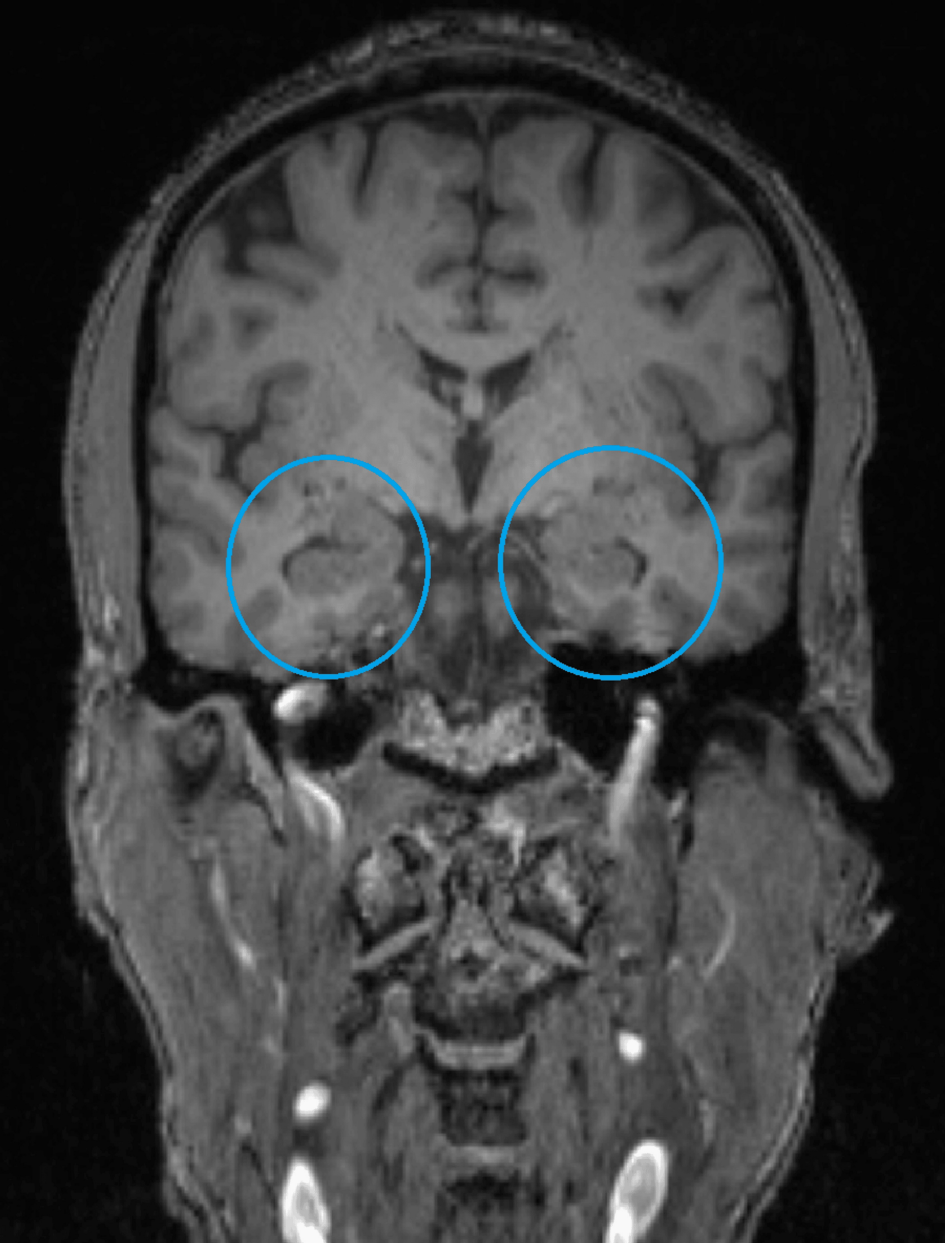
Coronal MRI No significant atrophy. Normal-sized hippocampus/entorhinal cortex volume (blue circle).

At the four-week follow-up, the patient presented alone and denied any mood or psychotic symptoms, and there was no change in memory symptoms. The brain MRI (Figures 1, 2) was reviewed by specialists in neurodegenerative diseases and showed no evidence suggestive of neurodegenerative pathology. Consequently, donepezil was increased to 10 mg daily, and a fluorodeoxyglucose-positron emission tomography (FDG-PET) scan was ordered to further assess for any neurodegenerative processes.

Two months later, the patient was again brought to the ED under an ex parte order by his daughter. He was medically cleared for psychiatric evaluation, and during the evaluation, he was found to be uncooperative, guarded, and exhibiting severe paranoia, refusing to engage in the interview. Collateral information from his daughter indicated that he had been noncompliant with his medications, leading to the reemergence of severe paranoia, confusion, aggressive behavior, and attempts to run away from home. The patient was readmitted to the inpatient unit, and his medications were reinstated, including donepezil 10 mg daily and olanzapine 2.5 mg nightly. He again responded well to the treatment, with daily improvements, and was discharged after seven days of hospitalization.

**Figure 3:**
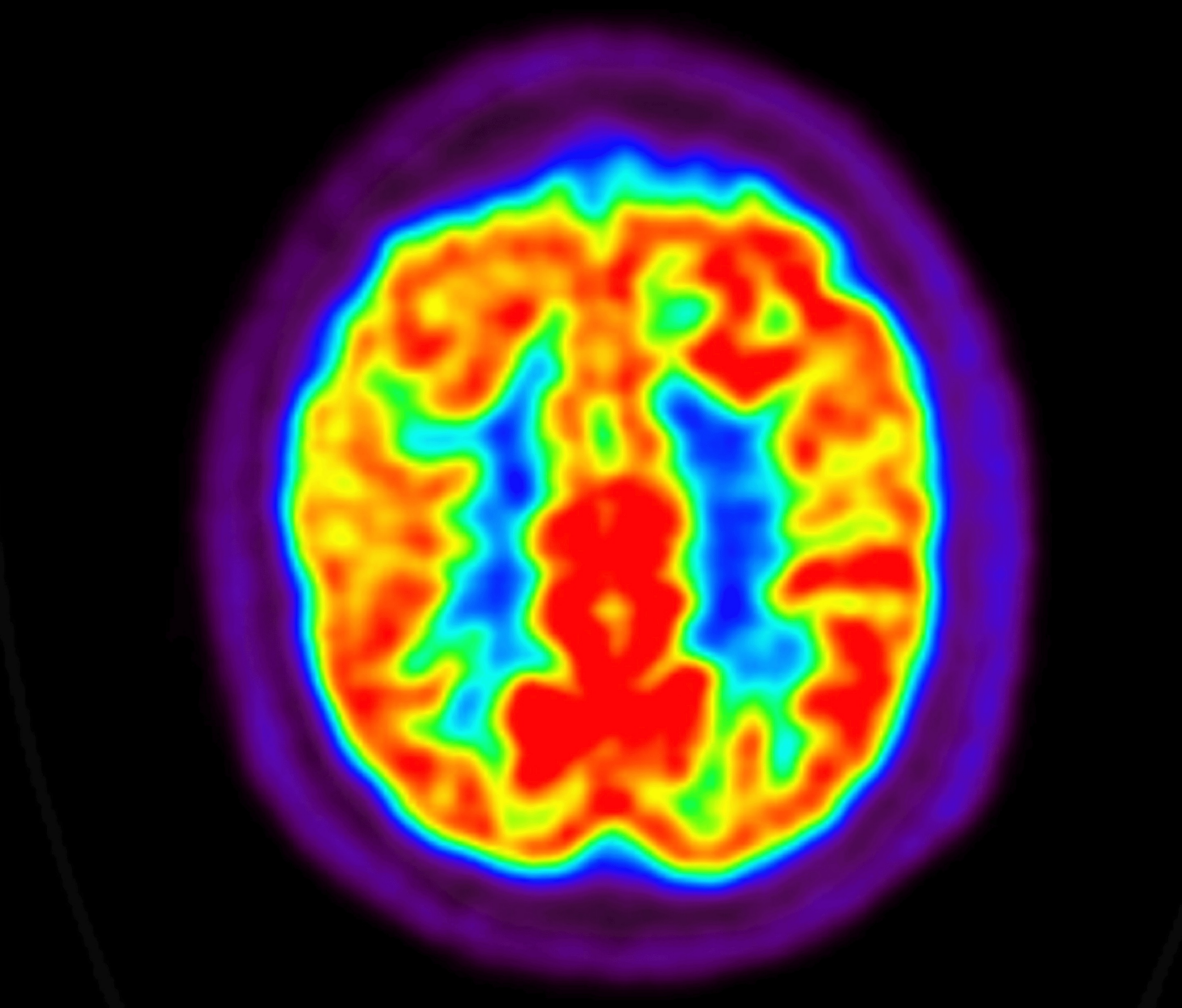
Fluorodeoxyglucose-positron emission tomography (FDG-PET) scan Normal metabolism of glucose.

The patient returned for a follow-up at the Wien Center one week post-discharge. During this visit, his daughter reported episodes of mood instability characterized by decreased need for sleep, increased energy, irritability, and periods of hypoverbality and reduced energy. The FDG-PET scan (Figure 3) was reviewed and was found to be within normal limits, showing no evidence of a neurodegenerative process. Based on these findings, donepezil was discontinued, and olanzapine was increased to 5 mg nightly. Additional investigations, including rapid plasma reagin (RPR), thyroid-stimulating hormone (TSH), and free thyroxine (T4) levels, were conducted and returned within normal limits.

The patient has had two subsequent follow-up visits, one alone and one accompanied by his daughter. He has remained compliant with olanzapine 5 mg nightly and has not exhibited any further symptoms of mood instability or psychosis.

## Discussion

This case highlights the diagnostic complexity and clinical challenges associated with late-onset psychiatric symptoms in elderly patients, particularly in distinguishing between primary psychiatric disorders and neurodegenerative conditions. The patient presented with symptoms consistent with very late-onset schizophrenia, including paranoia, persecutory delusions, and disorganized behavior, which persisted for over six months. Comprehensive evaluations, including neuroimaging and laboratory tests, were instrumental in ruling out neurodegenerative conditions and other medical causes, thus supporting a psychiatric diagnosis according to DSM-5 criteria.

The case underscores the diagnostic utility of advanced neuroimaging techniques, such as MRI and FDG-PET scans, in elderly patients with new-onset psychotic symptoms. While this imaging is primarily used to identify neurodegenerative diseases such as Alzheimer’s disease or dementia with Lewy bodies, its normal findings in this case contributed to excluding these conditions and optimizing treatment [5]. This aligns with existing literature emphasizing the role of imaging in differentiating psychiatric from neurodegenerative disorders but adds valuable data on its application in very late-onset schizophrenia, a relatively rare diagnosis [6].

Differentiating very late-onset schizophrenia from neurodegenerative conditions presents a significant diagnostic challenge due to overlapping symptomatology, including hallucinations, delusions, and behavioral disturbances. A systematic approach is essential, encompassing the exclusion of medical causes, longitudinal symptom assessment, and thorough collateral history. Advanced diagnostic modalities, such as dopamine transporter SPECT and myocardial scintigraphy, have demonstrated utility in distinguishing very late-onset schizophrenia (VLOS) from neurodegenerative diseases like dementia with Lewy bodies (DLB). Additionally, vascular factors, including white matter hyperintensities and hypertension, have been implicated in late-life psychosis, further complicating diagnostic precision [7]. Notably, MRI has shown diagnostic utility, with reported sensitivities of 90% for Alzheimer's disease and 78.7% for DLB, underscoring its role in enhancing diagnostic accuracy [8].

The presentation of mood symptoms, such as periods of elevated mood and decreased sleep, raised the possibility of alternative diagnoses like schizoaffective disorder or bipolar disorder with psychosis. However, the predominance of psychotic symptoms and absence of a clear mood episode pointed to schizophrenia as the most likely diagnosis [1,2].

The patient’s positive response to olanzapine monotherapy emphasizes the importance of targeted antipsychotic treatment in managing very late-onset schizophrenia. This case also illustrates the challenges of medication noncompliance, a common issue in the elderly, and the role of family involvement in ensuring adherence. Additionally, the temporary use of an acetylcholinesterase inhibitor, based on initial concerns for possible neurodegenerative processes, highlights the need for careful medication management to avoid unnecessary polypharmacy [3]. From a clinical perspective, this case underscores the importance of comprehensive evaluations that include not only neuroimaging but also detailed psychiatric assessments and longitudinal monitoring. Accurate differentiation between psychiatric and neurodegenerative conditions is critical to providing appropriate therapy and minimizing the risk of adverse outcomes.

## Conclusions

This case highlights the diagnostic complexity associated with late-onset psychiatric symptoms in elderly patients, particularly in distinguishing primary psychiatric disorders from neurodegenerative conditions. The integration of advanced neuroimaging with comprehensive clinical and psychiatric evaluations was instrumental in excluding dementia and establishing a diagnosis of very late-onset schizophrenia. The patient’s favorable response to antipsychotic monotherapy underscores the necessity of individualized treatment strategies in geriatric psychiatry, emphasizing both therapeutic efficacy and tolerability. Importantly, this case highlights the critical role of detailed history taking, particularly regarding symptom onset, progression, and associated risk factors, in conjunction with neuroimaging and treatment response to enhance diagnostic accuracy. Furthermore, it underscores the importance of long-term monitoring, caregiver engagement, and continued research into the neurobiological underpinnings and progression of late-onset psychotic disorders to refine diagnostic and therapeutic approaches.
